# Evaluating the Contribution of the Cause of Kidney Disease to Prognosis in CKD: Results From the Study of Heart and Renal Protection (SHARP)

**DOI:** 10.1053/j.ajkd.2013.12.013

**Published:** 2014-07

**Authors:** Richard Haynes, Natalie Staplin, Jonathan Emberson, William G. Herrington, Charles Tomson, Lawrence Agodoa, Vladimir Tesar, Adeera Levin, David Lewis, Christina Reith, Colin Baigent, Martin J. Landray, SHARP Collaborative Group

**Affiliations:** 1 Clinical Trial Service Unit and Epidemiological Studies Unit, Nuffield Department of Population Health, University of Oxford, Oxford, United Kingdom; 2 North Bristol NHS Trust, Bristol, United Kingdom; 3 National Institute of Diabetes and Digestive and Kidney Diseases, National Institutes of Health, Bethesda, MD; 4 First Faculty of Medicine and General University Hospital, Charles University, Prague, Czech Republic; 5 University of British Columbia, Vancouver, BC, Canada

**Keywords:** Kidney disease etiology, disease trajectory, end-stage renal disease (ESRD), disease progression, prognosis, cystic kidney disease, risk factor

## Abstract

**Background:**

The relevance of the cause of kidney disease to prognosis among patients with chronic kidney disease is uncertain.

**Study Design:**

Observational study.

**Settings & Participants:**

6,245 nondialysis participants in the Study of Heart and Renal Protection (SHARP).

**Predictor:**

Baseline cause of kidney disease was categorized into 4 groups: cystic kidney disease, diabetic nephropathy, glomerulonephritis, and other recorded diagnoses.

**Outcomes:**

End-stage renal disease (ESRD; dialysis or transplantation) and death.

**Results:**

During an average 4.7 years' follow-up, 2,080 participants progressed to ESRD, including 454 with cystic kidney disease (23% per year), 378 with glomerulonephritis (10% per year), 309 with diabetic nephropathy (12% per year), and 939 with other recorded diagnoses (8% per year). By comparison with patients with cystic kidney disease, other disease groups had substantially lower adjusted risks of ESRD (relative risks of 0.28 [95% CI, 0.24-0.32], 0.40 [95% CI, 0.34-0.47], and 0.29 [95% CI, 0.25-0.32] for glomerulonephritis, diabetic nephropathy, and other recorded diagnoses, respectively). Albuminuria and baseline estimated glomerular filtration rate were associated more weakly with risk of ESRD in patients with cystic kidney disease than the 3 other diagnostic categories (*P* for interaction, <0.001 and 0.01, respectively). Death before ESRD was uncommon in patients with cystic kidney disease, but was a major competing risk for participants with diabetic nephropathy, whose adjusted risk of death was 2-fold higher than that of the cystic kidney disease group (relative risk, 2.35 [95% CI, 1.73-3.18]).

**Limitations:**

Exclusion of patients with prior myocardial infarction or coronary revascularization.

**Conclusions:**

The cause of kidney disease has substantial prognostic implications. Other things being equal, patients with cystic kidney disease are at much higher risk of ESRD (and much lower risk of death before ESRD) than other patients. Patients with diabetic nephropathy are at particularly high risk of death prior to reaching ESRD.

Chronic kidney disease (CKD) is common and increasing in prevalence, largely due to population aging and an increasing incidence of diabetes mellitus.^1^ Recent studies have highlighted the prognostic relevance of measures of kidney function (eg, estimated glomerular filtration rate [eGFR]) and albuminuria (eg, urine albumin-creatinine ratio [ACR]).^2^, ^3^ Lower eGFR and higher ACR values are associated independently with increased risks of cardiovascular mortality, all-cause mortality, and end-stage renal disease (ESRD; ie, the need for dialysis or transplantation).^2^, ^3^, ^4^ Recent international guidelines recommend classifying CKD based on cause, eGFR, and ACR.^5^ However, it is unclear whether the underlying condition responsible for CKD (the cause of kidney disease) has independent prognostic significance for the risks of progression or death beyond that mediated by known risk factors (eg, albuminuria). Previous observational studies have been limited to assessments of the relevance of the cause of kidney disease to kidney disease progression,^6^, ^7^, ^8^, ^9^, ^10^, ^11^ whereas little consideration has been given to its effect on mortality. The largest such study, the MDRD (Modification of Diet in Renal Disease) Study, demonstrated that polycystic kidney disease resulted in more rapid progression than other primary kidney diseases.^10^, ^11^ However, this study excluded many patients with diabetic nephropathy,^12^ now the most common cause of ESRD.^1^

The Study of Heart and Renal Protection (SHARP) was a large randomized trial of the effects of cholesterol-lowering therapy on clinical outcomes. Detailed information for the cause of kidney disease, kidney function, and other risk factors at baseline, together with kidney function (including the need for renal replacement therapy) and death (including the adjudicated cause) during the 5 years of follow-up, allows the relevance of the cause of kidney disease to kidney disease progression and the risk of death to be explored in more detail.

## Methods

### Study Overview

The SHARP trial (ClinicalTrials.gov identifier NCT00125593; ISRCTN.org study number 54137607) investigated the efficacy of lowering low-density lipoprotein cholesterol levels with simvastatin (20 mg daily) plus ezetimibe (10 mg daily) in 9,270 participants with CKD (of whom 6,245 were not on dialysis therapy at study entry).^13^ The trial methods have been published in detail elsewhere and are summarized below.^13^, ^14^ Ethics approval was obtained from all study sites prior to enrollment.

### Recruitment and Eligibility Criteria

Individuals 40 years or older were eligible to participate if they had CKD with more than one previous measurement of serum or plasma creatinine of at least 1.7 mg/dL (150 μmol/L) in men or 1.5 mg/dL (130 μmol/L) in women. Participants with prior myocardial infarction or coronary revascularization were excluded.

### Baseline Assessment

Cause of kidney disease was recorded by trained study clinic staff prior to randomization (based on the managing physician's clinical diagnosis of the predominant cause of kidney disease, ie, the “primary renal diagnosis”) and subsequently categorized into 1 of 4 groups: glomerulonephritis, diabetic nephropathy, cystic kidney disease, and a group that included all other recorded diagnoses (including hypertension, renovascular disease, pyelonephritis, other known diagnoses, or unknown diagnosis). The grouping of these other recorded diagnoses into a single category was done on the basis that participants with such diagnoses had similar characteristics and prognosis (see Tables S1 and S2, available as online supplementary material).

The presence of prior vascular disease (coronary artery disease excluding myocardial infarction or revascularization, stroke, or peripheral arterial disease), diabetes, smoking status, race, and comedication were based on self-report by participants. At the randomization visit, blood pressure, height, and weight were measured and samples of nonfasting blood and urine were collected from all participants. Blood samples were cooled, centrifuged, and separated before being stored locally at −40°C. Samples then were shipped on dry ice to the central laboratory in Oxford, where assays of plasma creatinine and urinary ACR were conducted. Creatinine and albumin were measured using a Synchron LX20 or DXC800 analyzer (Beckman Coulter). Creatinine was assayed using a kinetic alkaline picrate method, calibrated using material traceable to National Institute of Standards and Technology (NIST) Standard Reference Material 914a, with a mean expanded uncertainty of 13.4% (7.3% excluding biological variation).

### Follow-up

After 6 weeks of placebo run-in, participants were randomly assigned to receive the main comparison of simvastatin (20 mg) plus ezetimibe (10 mg) as a single tablet versus matching placebo versus simvastatin (20 mg) alone in a ratio of 4:4:1, and treatment allocation was masked using a double-dummy method. After 1 year, patients initially allocated to receive simvastatin alone (and who were alive and willing to continue) were randomly assigned to receive simvastatin plus ezetimibe versus placebo. Participants were seen at 2 and 6 months after randomization and then every 6 months until final follow-up, on average 5 years after initial randomization. At each visit, blood samples were obtained and creatinine was analyzed in each site's local laboratory. Information for all serious adverse events was sought at each visit and further documentation was collected on events of interest (including initiation of renal replacement therapy and all deaths) by study staff. This information was sent to the international coordinating center for central adjudication, in accordance with prespecified definitions, by trained clinicians who remained blinded to study treatment allocation.

The 2 main outcomes of interest for these analyses were ESRD and death, separately. Rate of change in kidney function was calculated from local hospital creatinine measurements on samples obtained at the time of study visits (on average, 9 measurements per patient were available for slope estimation). eGFR was calculated using the 4-variable MDRD Study equation.^15^ Secondary outcomes included the composite outcomes of ESRD or death and ESRD or doubling of creatinine level.

### Statistical Analysis

Baseline characteristics that were identified as potential risk factors for ESRD and mortality were age, sex, country, race, treatment allocation, prior vascular disease, medication, lipid levels, smoking, blood pressure, body mass index (BMI), phosphate level, hemoglobin level, eGFR, and urinary ACR. Standard Cox regression techniques were used to assess the etiologic relevance of various baseline characteristics to risk.^16^

In multivariable analyses, lipid levels, blood pressure, BMI, phosphate level, hemoglobin level, eGFR, and ACR were fitted as categorical variables (including when necessary a category for missing values) to allow for any potential nonlinearity in the risk relationships. The categories used for these variables are as follows: diastolic blood pressure, <80, 80-89, 90-99, and ≥100 mm Hg; systolic blood pressure, <140, 140-159, 160-179, and ≥180 mm Hg; low-density lipoprotein cholesterol, <97, ≥97-<116, and ≥116 mg/dL (<2.5, ≥2.5-<3.0, and ≥3.0 mmol/L); high-density lipoprotein cholesterol, <39, ≥39-<46, and ≥46 mg/dL (<1.0, ≥1.0-<1.2, and ≥1.2 mmol/L); triglycerides, <133, ≥133-<177, and ≥177 mg/dL (<1.5, ≥1.5-<2.0, and ≥2.0 mmol/L); BMI, <24, ≥24-<28, and ≥28 kg/m^2^; phosphate, <3.7, ≥3.7-<4.6, and ≥4.6 mg/dL (<1.2, ≥1.2-<1.5, and ≥1.5 mmol/L); hemoglobin, <12, ≥12-<13, and ≥13 g/dL; eGFR, <15, ≥15-<30, ≥30-<60, and ≥60 mL/min/1.73 m^2^; and ACR, <30, ≥30-<300, and ≥300 mg/g.

In addition, because of the potential for the competing risk of death before ESRD, cumulative incidence functions were used to estimate how the risks of ESRD and death prior to ESRD actually emerged over time.^17^ Both Cox and, separately, Fine and Gray proportional hazards regression models were used to estimate the relevance of cause of kidney disease to the risks of ESRD and mortality, before and after adjustment for other risk factors.^16^, ^18^ Adjusted relative risks (RRs; approximated by the hazard ratios from the Cox models and the subdistribution HRs from the Fine and Gray models), together with their 95% confidence intervals (CIs), were estimated for each cause of kidney disease group relative to the group of participants with cystic kidney disease. In figures, these RRs are presented as “floating absolute risks”, which ascribes an appropriate variance to the log of the RR in every group (including the reference group with RR of 1.00), allowing comparisons to be made between any 2 groups.^19^ However, in the text, all quoted RRs comparing specified groups of participants are provided with the appropriate “unfloated” CI for that direct comparison.

To assess whether the prognostic relevance of urinary ACR and eGFR to ESRD risk differed depending on the cause of kidney disease, models with appropriate interaction terms were fitted. The proportional hazards assumption was tested through examination of the Schoenfeld partial residuals. To calculate the annual rate of change of eGFR for each patient, linear regression was used to estimate the rate of change in eGFR from local creatinine values (ignoring measurements after ESRD). The validity of making such a “linearity” assumption has been assessed previously and confirmed to be appropriate.^20^ Since the reliability of such estimated progression rates is affected strongly by the number of available creatinine values (with fewer creatinine measurements resulting in less reliable estimates of the true progression rate), participants with fewer than 3 follow-up creatinine measurements were excluded. In addition, participants for whom the mean deviation from their own fitted slope was in the top 1% of the distribution (of mean deviations across all participants) also were excluded (1,211 [20%] were excluded in total). Mixed models then were used to assess the amount of variation in progression rate explained by cause of kidney disease. These models adjusted for all other characteristics that were identified as possible risk factors for kidney disease progression, with the exception of baseline eGFR (because this was used in the calculation of the progression rate, which was the response variable in the mixed models).

## Results

Of 6,245 SHARP participants who were not on dialysis therapy at study entry, 5,990 had a recorded cause of kidney disease: 675 (11%) had cystic kidney disease, 1,049 (18%) had glomerulonephritis (most commonly, “glomerulonephritis–not histologically examined” [253 participants] or immunoglobulin A nephropathy [229 participants]) and 886 (15%) had diabetic nephropathy (Table 1). The group of 3,380 participants with other recorded diagnoses included 993 with hypertensive disease, 404 with pyelonephritis, 1,197 with other known diagnosis, and 786 with no known cause.

**Table 1:** Baseline Characteristics by Renal Diagnosis for 5,990 Patients Not Receiving Dialysis at Randomization and With a Classified Baseline Cause of Kidney Disease

	Cystic Kidney Disease (n = 675)	Glomerulonephritis (n = 1,049)	Diabetic Nephropathy (n = 886)	Other Recorded Diagnoses^a^ (n = 3,380)	*P* for Differences Between Diagnosis Groups
Age at randomization (y)^b^	56 ± 10	59 ± 12	64 ± 10	65 ± 12	<0.001
Men	360 (53)	653 (62)	565 (64)	2,130 (63)	<0.001
Prior vascular disease^b^	50 (7)	85 (8)	221 (25)	539 (16)	<0.001
Diabetes^b^	25 (4)	80 (8)	886 (100)	398 (12)	<0.001
Current smoker^b^	94 (14)	121 (12)	90 (10)	429 (13)	0.09
Systolic BP (mm Hg)^b^	137 ± 18	136 ± 19	145 ± 22	139 ± 21	<0.001
Diastolic BP (mm Hg)^b^	84 ± 11	81 ± 12	76 ± 12	80 ± 12	<0.001
Apolipoprotein A1 (mg/dL)^b^	137.11 ± 26.43	138.47 ± 29.56	130.83 ± 28.20	137.21 ± 29.35	<0.001
Apolipoprotein B (mg/dL)^b^	96.10 ± 21.65	102.01 ± 25.81	96.66 ± 27.74	98.63 ± 25.13	<0.001
Phosphate (mmol/L)	1.30 ± 0.32	1.28 ± 0.33	1.32 ± 0.35	1.23 ± 0.30	<0.001
Hemoglobin (g/dL)	12.59 ± 1.53	12.54 ± 1.73	12.04 ± 1.71	12.68 ± 1.70	<0.001
BMI (kg/m²)^b^	26.8 ± 4.6	26.8 ± 5.2	28.3 ± 6.2	27.4 ± 5.4	<0.001
Race					<0.001
White	597 (88)	763 (73)	435 (49)	2,473 (73)	
Black	4 (<1)	3 (<1)	18 (2)	86 (3)	
Asian	58 (9)	256 (24)	419 (47)	759 (22)	
Other/not specified	16 (2)	27 (3)	14 (2)	62 (2)	
Comedication^b^					
Antiplatelet therapy	61 (9)	147 (14)	276 (31)	704 (21)	<0.001
ACEi or ARB	499 (74)	716 (68)	566 (64)	1,895 (56)	<0.001
β-Blocker	267 (40)	337 (32)	336 (38)	1296 (38)	0.002
Calcium channel blocker	308 (46)	416 (40)	451 (51)	1,460 (43)	<0.001
eGFR (mL/min/1.73 m^2^)^b^^c^					
Mean	22.8 ± 11.1	25.7 ± 12.4	27.6 ± 14.6	27.2 ± 13.1	<0.001
<15	193 (30)	225 (22)	170 (20)	593 (18)	
≥15-<30	277 (43)	417 (41)	362 (42)	1,399 (43)	
≥30-<60	174 (27)	371 (36)	311 (36)	1,202 (37)	
≥60	1 (0)	9 (1)	21 (2)	55 (2)	
Urinary ACR (mg/g)^c^					
Median	102 [36-265]	436 [138-1,074]	601 [137-2,024]	143 [30-584]	<0.001
<30	126 (20)	92 (9)	91 (11)	744 (25)	
≥30-<300	354 (57)	310 (32)	208 (26)	1,143 (38)	
≥300	138 (22)	571 (59)	498 (62)	1,083 (36)	
Randomized to simvastatin + ezetimibe	329 (49)	528 (50)	445 (50)	1,696 (50)	0.9

*Note:* Values for categorical variables are given as number (percentage); values for continuous variables, as mean ± standard deviation or median [interquartile range]. There were 6,245 patients not on dialysis therapy at randomization, but 255 had missing values for renal diagnosis and have been excluded from all further analyses.

Abbreviations: ACEi, angiotensin-converting enzyme inhibitor; ACR, albumin-creatinine ratio; ARB, angiotensin receptor blocker; BMI, body mass index; BP, blood pressure; eGFR, estimated glomerular filtration rate.

a The group of 3,380 participants with “other recorded diagnoses” included 993 with hypertensive disease, 404 with pyelonephritis, 1,197 with other known diagnosis, and 786 with no known cause.

b Variables updated at 1 year for patients originally allocated to simvastatin only who were re–randomly assigned to simvastatin plus ezetimibe or placebo.

c Percentages exclude participants for whom data were not available for that category.

Participants with cystic kidney disease were younger and had lower eGFRs at study entry than the other groups (Table 1). Average baseline ACRs in the cystic kidney disease and combined other recorded diagnoses groups were broadly similar (102 and 143 mg/g, respectively) but were substantially higher in the glomerulonephritis (436 mg/g) and diabetic nephropathy (601 mg/g) groups. Participants with diabetic nephropathy were more likely to have prior vascular disease (25%) than participants with glomerulonephritis (8%), cystic kidney disease (7%), or other recorded diagnoses (16%) and more often were of Asian origin (47% vs 24%, 9%, and 22% respectively).

For the 5,990 participants not on dialysis therapy at study entry and with a recorded cause of kidney disease, average follow-up was 4.7 years. The mean annual change in eGFR was fastest for participants with cystic kidney disease (−3.8 mL/min/1.73 m^2^ per year), intermediate for participants with glomerulonephritis (−1.9 mL/min/1.73 m^2^ per year) or diabetic nephropathy (−2.5 mL/min/1.73 m^2^ per year), and slowest for participants with other recorded diagnoses (−1.2 mL/min/1.73 m^2^ per year; Table 2). In the group of 3,380 participants with other recorded diagnoses, mean annual rates of change in eGFR were broadly similar for each of the main subcategories (Table S2). Overall, primary kidney disease explained 16% of the variation in rate of change of eGFR not already explained by other measured prognostic factors. Sensitivity analysis including all patients in the calculation of mean annual change in eGFR did not materially change these estimates.

**Table 2:** Renal Progression by Renal Diagnosis in 5,990 Patients Not Receiving Dialysis at Randomization and With a Classified Baseline Cause of Kidney Disease

	Cystic Kidney Disease	Glomerulonephritis	Diabetic Nephropathy	Other Recorded Diagnoses
No. randomly assigned	675	1,049	886	3,380
Total person-years at risk of ESRD	1,942	3,711	2,536	12,145
Mean annual rate of change in eGFR (mL/min/1.73 m^2^ per y)	−3.8 ± 2.5	−1.9 ± 3.6	−2.5 ± 4.8	−1.2 ± 3.2
Excluded from calculation of mean annual rate of change in eGFR^a^	164 (24)	190 (18)	244 (28)	613 (18)
No. of first events				
ESRD	454 (23)	378 (10)	309 (12)	939 (8)
Death before ESRD	21 (1)	97 (3)	206 (8)	478 (4)
ESRD or death	475 (24)	475 (13)	514 (20)	1,417 (12)
Any death	57 (3)	154 (4)	315 (12)	687 (6)

*Note:* Unless otherwise indicated, values given as number (percentage) or mean ± standard deviation.

Abbreviations: eGFR, estimated glomerular filtration rate; ESRD, end-stage renal disease.

a Patients with fewer than 3 follow-up creatinine measurements or those with “poorly fitting slopes” (see methods) were excluded.

**Figure 1:**
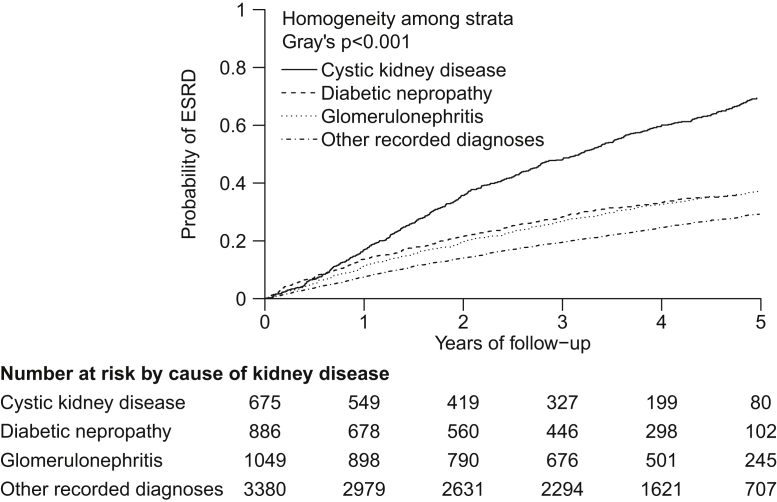
Cumulative incidence curves for end-stage renal disease (ESRD) by cause of kidney disease. These cumulative incidence curves account for the competing risk of death.

**Figure 2:**
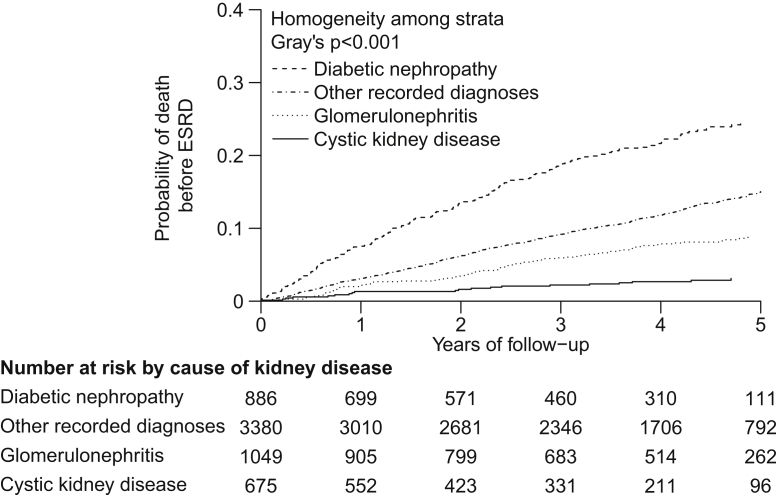
Cumulative incidence curves for death from any cause before progression to end-stage renal disease (ESRD), by cause of kidney disease. These cumulative incidence curves account for the competing risk of ESRD.

Overall, 2,080 participants reached ESRD. The unadjusted annual rate of reaching ESRD was about twice as high for participants with cystic kidney disease (23%) than for those with other primary renal diagnoses (glomerulonephritis, 10%; diabetic nephropathy, 12%; other recorded diagnoses, 8%; Table 2; Fig 1). By contrast, participants with cystic kidney disease had the lowest risk of death before ESRD (1%), participants with glomerulonephritis and those with other recorded diagnoses had intermediate risk (3% and 4%, respectively), and those with diabetic nephropathy had the highest risk (8%; Table 2; Fig 2). As a consequence, the overall risk of ESRD or death (before ESRD) was almost twice as high for participants with cystic kidney disease (24%) and diabetic nephropathy (20%) compared with other primary renal diagnoses (glomerulonephritis, 13%; other recorded diagnoses, 12%; Table 2; Fig S1).

**Figure 3:**
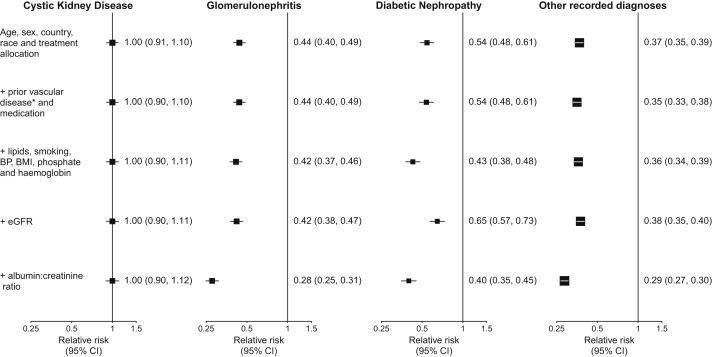
Effect of adjustment for known risk factors on the association between cause of kidney disease and end-stage renal disease, estimated using Cox regression. ^∗^Additional adjustment for prior diabetes has very little effect on the relative risks observed, but because the interpretation of the relative risk for the diabetic nephropathy group after adjustment for diabetes is unclear, it is not adjusted for in these analyses. Abbreviations: BMI, body mass index; BP, blood pressure; CI, confidence interval; eGFR, estimated glomerular filtration rate.

Figure 3 shows the effect of adjustment for baseline characteristics on the association between cause of kidney disease and ESRD. With the reference group being those having cystic kidney disease, the risk of ESRD (estimated from a basic model adjusted for only age, sex, country, race, and randomized treatment allocation) was only about half as big for participants with glomerulonephritis or diabetic nephropathy (RRs of 0.44 [95% CI for direct comparison, 0.38-0.51] and 0.54 [95% CI, 0.46-0.64], respectively), whereas the risk for those with other recorded diagnoses was nearly two-thirds lower than that seen in the cystic kidney disease group (RR, 0.37 [95% CI, 0.33-0.42]). Further adjustment for prior vascular disease, medication, lipid levels, smoking status, blood pressure, BMI, and eGFR did not change these estimates much, but subsequent additional adjustment for urinary ACR resulted in still bigger RR differences being observed between participants with cystic kidney disease and participants with other diagnoses. Considering those having cystic kidney disease as the reference group, the fully adjusted RRs for ESRD were 0.28 (95% CI for direct comparison, 0.24-0.32) for participants with glomerulonephritis, 0.40 (95% CI, 0.34-0.47) for those with diabetic nephropathy, and 0.29 (95% CI, 0.25-0.32) for those with other recorded diagnoses. Further subdivision of the group of participants with other recorded causes showed similar RRs for each subgroup (RRs of 0.29 [95% CI, 0.25-0.35], 0.24 [95% CI, 0.20-0.30], 0.30 [95% CI, 0.26-0.35], and 0.28 [95% CI, 0.24-0.34] for participants with hypertensive kidney disease, pyelonephritis, other known cause, and recorded cause unknown, respectively).

On the whole, RR estimates derived using Fine and Gray regression models were broadly similar to those derived from the Cox regression models (Fig 3; Fig S2). However, differences in ESRD risk between participants with diabetic nephropathy and those with cystic kidney disease became more pronounced when based on the Fine and Gray model rather than the Cox model (fully adjusted RRs for diabetic nephropathy vs cystic kidney disease were 0.33 [95% CI for direct comparison, 0.27-0.41] and 0.40 [95% CI, 0.34-0.47], respectively). This was because death before ESRD was a strong competing risk for patients with diabetic nephropathy. As a consequence, the actual rate at which patients with diabetic nephropathy would be expected to present with ESRD (as reflected by the Fine and Gray estimate) would be somewhat lower than would have been the case in the hypothetical absence of deaths before ESRD (as reflected by the Cox estimates). Thus, the difference in ESRD rates between the 2 groups becomes somewhat bigger when that competing risk is taken into account. Analogous findings were observed in a model of associations between primary kidney disease and mean annual decrease in eGFR (Table S3) and in analyses excluding participants with diabetes from the other recorded diagnoses group (data not shown).

eGFR was a highly significant predictor of the risks of ESRD for all categories of primary kidney disease, but there was some evidence that it was relatively less important in patients with cystic kidney disease (*P* for interaction = 0.01; Table 3). The relative effects of albuminuria on ESRD also varied according to the primary kidney disease (*P* for interaction < 0.001). For patients with cystic kidney disease, the risk of ESRD was not significantly higher in the presence of microalbuminuria (ACR of 30-300 mg/g: RR, 1.18; 95% CI, 0.88-1.58) or macroalbuminuria (ACR ≥ 300 mg/g: RR, 1.21; 95% CI, 0.87-1.69), whereas the risks of ESRD were all substantially increased in association with macroalbuminuria among patients with other primary kidney diseases (Table 3). This interaction between renal diagnosis and ACR was confirmed when the statistically more sensitive annual rate of change of eGFR was used as the outcome (data not shown). Although higher ACR predicted a greater annual decline in eGFR in all diagnoses, the association was weaker for patients with cystic kidney disease.

**Table 3:** Effect of ACR and eGFR Group on Progression to ESRD by Cause of Kidney Disease

	Cystic Kidney Disease	Glomerulonephritis	Diabetic Nephropathy	Other Recorded Diagnoses
Urinary ACR (mg/g)^a^				
<30	1.00 (reference)	1.00 (reference)	1.00 (reference)	1.00 (reference)
≥30-<300	1.18 (0.88-1.58)	2.39 (1.03-5.54)	1.64 (0.79-3.41)	1.39 (1.05-1.84)
≥300	1.21 (0.87-1.69)	7.26 (3.22-16.36)	5.85 (2.98-11.49)	3.91 (2.99-5.10)
eGFR (mL/min/1.73 m^2^)^b^				
<15	13.11 (9.13-18.82)	20.41 (13.98-29.79)	20.58 (14.07-30.11)	23.56 (17.95-30.92)
≥15-<30	4.81 (3.41-6.80)	4.71 (3.24-6.84)	3.95 (2.74-5.68)	5.13 (3.94-6.68)
≥30	1.00 (reference)	1.00 (reference)	1.00 (reference)	1.00 (reference)

*Note:* Values shown are relative risk (95% confidence interval).

Abbreviations: ACR, albumin-creatinine ratio; eGFR, estimated glomerular filtration rate; ESRD, end-stage renal disease.

a Interactions between albuminuria group and diagnoses of cystic kidney disease, glomerulonephritis, and diabetic nephropathy, after adjustment for age, sex, country, race, treatment allocation, prior diseases and medication, lipid levels, smoking, blood pressure, body mass index, phosphate level, hemoglobin level, and eGFR.

b Interactions between eGFR group and diagnoses of cystic kidney disease, glomerulonephritis, and diabetic nephropathy, after adjustment for age, sex, country, race, treatment allocation, prior diseases and medication, lipid levels, smoking, blood pressure, body mass index, phosphate level, hemoglobin level, and ACR.

**Figure 4:**
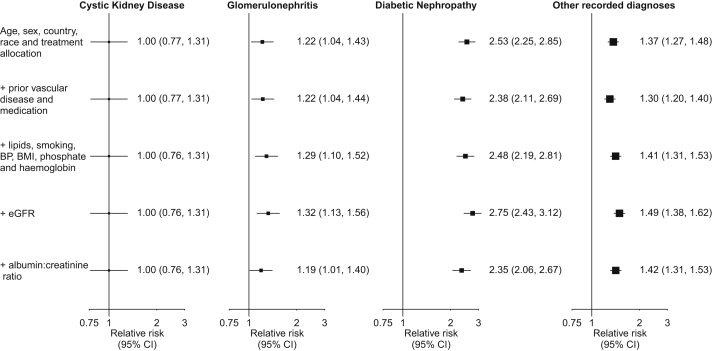
Effect of adjustment for known risk factors on the association between cause of kidney disease and death at any time, estimated using Cox regression. Abbreviations: BMI, body mass index; BP, blood pressure; CI, confidence interval; eGFR, estimated glomerular filtration rate.

Figure 4 shows the effect of adjustment for baseline characteristics on the association between cause of kidney disease and death. Other things being equal, participants with cystic kidney disease had the lowest mortality rates. Compared with participants with cystic kidney disease, mortality rates for participants with diabetic nephropathy were more than twice as high (fully adjusted RR, 2.35; 95% CI for direct comparison, 1.73-3.18), mortality rates for participants with glomerulonephritis were about one-fifth higher (RR, 1.19; 95% CI, 0.87-1.63), and mortality rates for participants with other recorded diagnoses were about two-fifths higher (RR, 1.42; 95% CI, 1.07-1.87).

## Discussion

These analyses show that after adjusting for differences in prognostic factors, cystic kidney disease was associated with a 3-fold higher risk of ESRD and at least a one-third lower risk of death compared with other primary kidney diseases. Polycystic kidney disease, which is the main cystic disease leading to ESRD, is a tubular disease that progresses due to the genetically determined inexorable development and enlargement of cysts that gradually decrease function of the surrounding renal tissue, and this may explain why adjustment for known risk factors did not attenuate the association between cystic kidney disease and ESRD risk. Albuminuria in particular was not associated significantly with increased risk of ESRD (and only weakly associated with rate of decline in eGFR) for patients with cystic kidney disease, whereas it was a strong predictor of ESRD risk (and rate of decline in eGFR) for participants with other (chiefly glomerular) primary kidney diseases (Table 3). By contrast, the lower risk of death in association with cystic kidney disease, which remained after adjustment for prognostic risk factors, is of uncertain clinical significance and plausibly could be explained by residual confounding (such as unmeasured comorbid conditions).

Our analysis also demonstrated clearly that after adjustment for albuminuria and other known risk factors, participants in SHARP with glomerulonephritis had risks of progression to ESRD similar to participants with other (noncystic) primary renal diagnoses. Similarly, the risk of death for participants with glomerulonephritis was intermediate between the diabetic nephropathy group (who were at highest risk) and the cystic kidney disease group (at lowest risk) and similar to the group of participants with other recorded diagnoses.

The difference between the standard Cox model and Fine and Gray model (which accounts for competing risks and aims to estimate the prognostic impact of exposures on outcomes) was apparent only for participants with diabetic nephropathy. Because participants with diabetic nephropathy were much more likely than other participants to die before reaching ESRD, the ESRD rate that would have been observed in this group (other things being equal and in a population similar to that recruited into SHARP) would be similar to the rate observed in other participants without cystic kidney disease. This is reflected by the similar RRs for ESRD from the Fine and Gray model for participants with diabetic nephropathy and those with glomerulonephritis (RRs of 0.33 [95% CI, 0.27-0.41] and 0.32 [95% CI, 0.27-0.38], respectively) compared with participants with cystic kidney disease, whereas estimated RRs were significantly different in the Cox model (RRs of 0.40 [95% CI, 0.34-0.47] and 0.28 [95% CI, 0.24-0.32], respectively). In the SHARP population, participants with diabetic nephropathy had a similar probability of reaching ESRD or dying beforehand. However, it is important to recognize that patients with known coronary heart disease were excluded in SHARP, whereas about a quarter of all patients with diabetic nephropathy have a history of coronary heart disease.^21^, ^22^ For this reason, the risks of dying before reaching ESRD among unselected patients with diabetic nephropathy are likely to be even greater than observed in SHARP and probably would be greater than the risk of commencing dialysis therapy. Although measures to delay progression of kidney disease are important, this finding emphasizes the importance of vascular risk (the most common cause of death in this population) management for such patients.

The SHARP study population was made up of willing participants selected for inclusion into a randomized trial. Consequently, they are not likely to be representative of the CKD population as a whole. However, the estimates of relative differences presented (at least from the Cox regression models) still should be generalizable to other populations with CKD, as supported by the consistency between our findings and those from previous, admittedly smaller, CKD cohorts.^11^ Nearly all participants in SHARP had CKD stage 3b or worse, so results may not be generalizable to less severe stages of CKD, but most patients followed up in specialist nephrology clinics have degrees of CKD similar to those of the SHARP population.

In conclusion, the cause of kidney disease has substantial prognostic implications that persist when other prognostic factors are taken into account. Patients with cystic kidney disease are at much higher risk of ESRD (and much lower risk of death) than other patients. By contrast, patients with diabetic nephropathy are at particularly high risk of death before reaching ESRD.

## Supplementary material

Supplementary Table S1 (PDF) Baseline characteristics by kidney disease cause, in patients not on dialysis at randomization and with baseline cause.

Supplementary Table S2 (PDF) Renal progression by kidney disease cause, in patients not on dialysis at randomization and with baseline cause.

Supplementary Table S3 (PDF) Effect of adjustment for known risk factors on mean annual eGFR decrease by cause of kidney disease.

Supplementary Figure S1 (PDF) Life table plot for ESRD or death, by cause of kidney disease.

Supplementary Figure S2 (PDF) Effect of adjustment for known risk factors on association between cause of kidney disease and ESRD.
