# Supplementary material for: Evaluating the Contribution of the Cause of Kidney Disease to Prognosis in CKD: Results From the Study of Heart and Renal Protection (SHARP)
Source: Am J Kidney Dis. 2014 Jul;64(1):40–8. doi: 10.1053/j.ajkd.2013.12.013 (PMC4068325; doi:10.1053/j.ajkd.2013.12.013)
Supplement: Supplementary Table S1 (PDF) — Baseline characteristics by kidney disease cause, in patients not on dialysis at randomization and with baseline cause. [file mmc1.pdf]

Table S1: Baseline characteristics by cause of kidney disease, among 5990 patients not on dialysis at randomisation and with a classified baseline cause of renal disease

				Other recorded diagnoses				
	**Cystic kidney disease (n=675)**	**Glomerulonephritis (n=1049)**	**Diabetic nephropathy (n=886)**	**Hypertensive (n=993)**	**Pyelonephritis (n=404)**	**Other known diagnosis (n=1197)**	**Classified as 'unknown' (n=786)**	**Other recorded diagnoses subtotal (n=3380)**
Age at randomisation (years)*	56 (10)	59 (12)	64 (10)	65 (12)	63 (12)	65 (11)	66 (12)	65 (12)
Men	360 (53%)	653 (62%)	565 (64%)	647 (65%)	210 (52%)	771 (64%)	502 (64%)	2130 (63%)
Prior vascular disease*	50 (7%)	85 (8%)	221 (25%)	159 (16%)	44 (11%)	214 (18%)	122 (16%)	539 (16%)
Diabetes*	25 (4%)	80 (8%)	886 (100%)	139 (14%)	40 (10%)	128 (11%)	91 (12%)	398 (12%)
Current smoker*	94 (14%)	121 (12%)	90 (10%)	138 (14%)	51 (13%)	159 (13%)	81 (10%)	429 (13%)
Diastolic blood pressure (mm Hg)*	84 (11)	81 (12)	76 (12)	80 (13)	81 (12)	79 (12)	79 (13)	80 (12)
Systolic blood pressure (mm Hg)*	137 (18)	136 (19)	145 (22)	142 (21)	139 (21)	138 (21)	138 (22)	139 (21)
Apolipoprotein A1 (mg/dL)*	137.11 (26.43)	138.47 (29.56)	130.83 (28.20)	136.42 (27.86)	141.15 (30.17)	136.80 (30.40)	136.76 (29.05)	137.21 (29.35)
Apolipoprotein B (mg/dL)*	96.10 (21.65)	102.01 (25.81)	96.66 (27.74)	99.90 (25.13)	99.43 (26.34)	97.44 (25.23)	98.39 (24.28)	98.63 (25.13)
Phosphate (mmol/L)	1.30 (0.32)	1.28 (0.33)	1.32 (0.35)	1.23 (0.30)	1.26 (0.29)	1.23 (0.31)	1.22 (0.30)	1.23 (0.30)
Haemoglobin (g/dL)†	12.59 (1.53)	12.54 (1.73)	12.04 (1.71)	12.64 (1.74)	12.76 (1.61)	12.68 (1.67)	12.68 (1.72)	12.68 (1.70)
Body mass index (kg/m²)*	26.8 (4.6)	26.8 (5.2)	28.3 (6.2)	27.5 (5.2)	27.2 (5.5)	27.3 (5.3)	27.3 (5.8)	27.4 (5.4)
Race								
White	597 (88%)	763 (73%)	435 (49%)	614 (62%)	327 (81%)	911 (76%)	621 (79%)	2473 (73%)
Black	4 (<1%)	3 (<1%)	18 (2%)	39 (4%)	0 (0%)	27 (2%)	20 (3%)	86 (3%)
Asian	58 (9%)	256 (24%)	419 (47%)	321 (32%)	74 (18%)	232 (19%)	132 (17%)	759 (22%)
Other/not specified	16 (2%)	27 (3%)	14 (2%)	19 (2%)	3 (<1%)	27 (2%)	13 (2%)	62 (2%)
Co-medication*								
Antiplatelet therapy	61 (9%)	147 (14%)	276 (31%)	237 (24%)	65 (16%)	237 (20%)	165 (21%)	704 (21%)
ACE inhibitor or ARB	499 (74%)	716 (68%)	566 (64%)	665 (67%)	200 (50%)	607 (51%)	423 (54%)	1895 (56%)
Beta blocker	267 (40%)	337 (32%)	336 (38%)	467 (47%)	139 (34%)	394 (33%)	296 (38%)	1296 (38%)
Calcium channel blocker	308 (46%)	416 (40%)	451 (51%)	544 (55%)	143 (35%)	442 (37%)	331 (42%)	1460 (43%)
MDRD-estimated GFR (mL/min/1.73m²)*†								
Mean (SD)	22.8 (11.1)	25.7 (12.4)	27.6 (14.6)	28.3 (14.0)	24.8 (11.6)	27.2 (13.4)	27.2 (11.8)	27.2 (13.1)
≥60	1 (0%)	9 (1%)	21 (2%)	20 (2%)	4 (1%)	23 (2%)	8 (1%)	55 (2%)
≥30 to <60	174 (27%)	371 (36%)	311 (36%)	376 (39%)	111 (28%)	425 (37%)	290 (39%)	1202 (37%)
≥15 to <30	277 (43%)	417 (41%)	362 (42%)	398 (41%)	189 (48%)	488 (43%)	324 (43%)	1399 (43%)
<15	193 (30%)	225 (22%)	170 (20%)	166 (17%)	89 (23%)	209 (18%)	129 (17%)	593 (18%)
Not available	30	27	22	33	11	52	35	131
Urinary albumin:creatinine ratio (mg/g) †								
Median (IQR)	102 (36-265)	436 (138-1074)	601 (137-2024)	180 (30-689)	198 (62-697)	123 (29-520)	125 (23-521)	143 (30-584)
<30	126 (20%)	92 (9%)	91 (11%)	221 (25%)	60 (16%)	269 (26%)	194 (28%)	744 (25%)
≥30 to ≤300	354 (57%)	310 (32%)	208 (26%)	310 (35%)	157 (42%)	415 (40%)	261 (38%)	1143 (38%)
>300	138 (22%)	571 (59%)	498 (62%)	348 (40%)	154 (42%)	355 (34%)	226 (33%)	1083 (36%)
Not available	57	76	89	114	33	158	105	410
Randomised to simvastatin plus ezetimibe	329 (49%)	528 (50%)	445 (50%)	492 (50%)	198 (49%)	602 (50%)	404 (51%)	1696 (50%)

Data are n (%), mean (SD), or median (IQR). There were 6245 patients not on dialysis at randomisation, but 255 had missing values for renal diagnosis and so have been excluded from all further analyses. *Variables updated at 1 year for patients originally allocated simvastatin only who were rerandomised to simvastatin plus ezetimibe or placebo. †Percentages exclude participants for whom data were not available for that category.
