# Supplementary material for: Evaluating the Contribution of the Cause of Kidney Disease to Prognosis in CKD: Results From the Study of Heart and Renal Protection (SHARP)
Source: Am J Kidney Dis. 2014 Jul;64(1):40–8. doi: 10.1053/j.ajkd.2013.12.013 (PMC4068325; doi:10.1053/j.ajkd.2013.12.013)
Supplement: Supplementary Table S2 (PDF) — Renal progression by kidney disease cause, in patients not on dialysis at randomization and with baseline cause. [file mmc2.pdf]

Table S2: Renal progression by cause of kidney disease, among 5990 patients not on dialysis at randomisation and with a classified baseline cause of renal disease

				Other recorded diagnoses				
	**Cystic kidney disease**	**Glomerulonephritis**	**Diabetic nephropathy**	**Hypertensive**	**Pyelonephritis**	**Other known diagnosis**	**Classfied as 'unknown'**	**Other recorded diagnoses subtotal**
Number randomised	675	1049	886	993	404	1197	786	3380
Total person years for ESRD	1942	3711	2536	3541	1504	4238	2862	12145
Mean annual rate of change in eGFR (SD)	-3.8 (2.5)	-1.9 (3.6)	-2.5 (4.8)	-1.4 (3.4)	-0.9 (2.3)	-1.1 (3.3)	-1.2 (3.0)	-1.2 (3.2)
Excluded from calculation of mean annual rate of change in eGFR*	164 (24%)	190 (18%)	244 (28%)	175 (18%)	60 (15%)	252 (21%)	126 (16%)	613 (18%)
Number of first events (% per year)								
ESRD	454 (23%)	378 (10%)	309 (12%)	277 (8%)	122 (8%)	329 (8%)	211 (7%)	939 (8%)
Death before ESRD	21 (1%)	97 (3%)	206 (8%)	136 (4%)	42 (3%)	186 (4%)	114 (4%)	478 (4%)
ESRD or death	475 (24%)	475 (13%)	514 (20%)	413 (12%)	164 (11%)	515 (12%)	325 (11%)	1417 (12%)
Any death	57 (3%)	154 (4%)	315 (12%)	197 (6%)	65 (4%)	254 (6%)	171 (6%)	687 (6%)

*Patients with fewer than three follow-up creatinine measurements or those with "poorly fitting slopes" (see methods) were excluded.
