# Supplementary material for: Evaluating the Contribution of the Cause of Kidney Disease to Prognosis in CKD: Results From the Study of Heart and Renal Protection (SHARP)
Source: Am J Kidney Dis. 2014 Jul;64(1):40–8. doi: 10.1053/j.ajkd.2013.12.013 (PMC4068325; doi:10.1053/j.ajkd.2013.12.013)
Supplement: Supplementary Table S3 (PDF) — Effect of adjustment for known risk factors on mean annual eGFR decrease by cause of kidney disease. [file mmc3.pdf]

Table S3: Effect of adjustment for known risk factors on mean annual decrease in eGFR by cause of kidney disease

	Mean annual decrease in eGFR (mL/min/1.73m²/year)			
	**Cystic kidney disease (n=511)**	**Glomerulonephritis (n=859)**	**Diabetic nephropathy (n=642)**	**Other recorded diagnoses (n=2767)**
Unadjusted model	3.77 (3.47-4.07)	1.88 (1.64-2.11)	2.52 (2.25-2.79)	1.19 (1.06-1.32)
+ age, sex, country, race and treatment allocation	3.74 (3.43-4.05)	1.77 (1.54-2.00)	2.32 (2.05-2.59)	1.28 (1.15-1.40)
+ prior vascular disease and medication	3.74 (3.43-4.05)	1.77 (1.53-2.00)	2.33 (2.06-2.61)	1.27 (1.14-1.40)
+ lipids and smoking	3.77 (3.46-4.07)	1.75 (1.52-1.99)	2.34 (2.07-2.61)	1.27 (1.14-1.40)
+ blood pressure and BMI	3.70 (3.39-4.01)	1.77 (1.54-2.00)	2.33 (2.05-2.61)	1.28 (1.15-1.41)
+ phosphate and haemoglobin	3.69 (3.38-3.99)	1.76 (1.53-1.99)	2.24 (1.96-2.51)	1.31 (1.18-1.44)
+ albumin:creatinine ratio	4.19 (3.89-4.48)	1.41 (1.19-1.63)	1.84 (1.58-2.10)	1.42 (1.30-1.54)

*These values based on estimated rates of change in eGFR for 4779 of the 5990 patients not on dialysis at randomisation and with a classified baseline cause of renal disease, 1161 patients with fewer than three follow-up creatinine measurements and a further 50 with "poorly fitting slopes" (see methods) were excluded.
