# Supplementary material for: Evaluating the Contribution of the Cause of Kidney Disease to Prognosis in CKD: Results From the Study of Heart and Renal Protection (SHARP)
Source: Am J Kidney Dis. 2014 Jul;64(1):40–8. doi: 10.1053/j.ajkd.2013.12.013 (PMC4068325; doi:10.1053/j.ajkd.2013.12.013)
Supplement: Supplementary Figure S1 (PDF) — Life table plot for ESRD or death, by cause of kidney disease. [file mmc4.pdf]

Figure S1: Life table plot for end stage renal disease or death, by cause of kidney disease

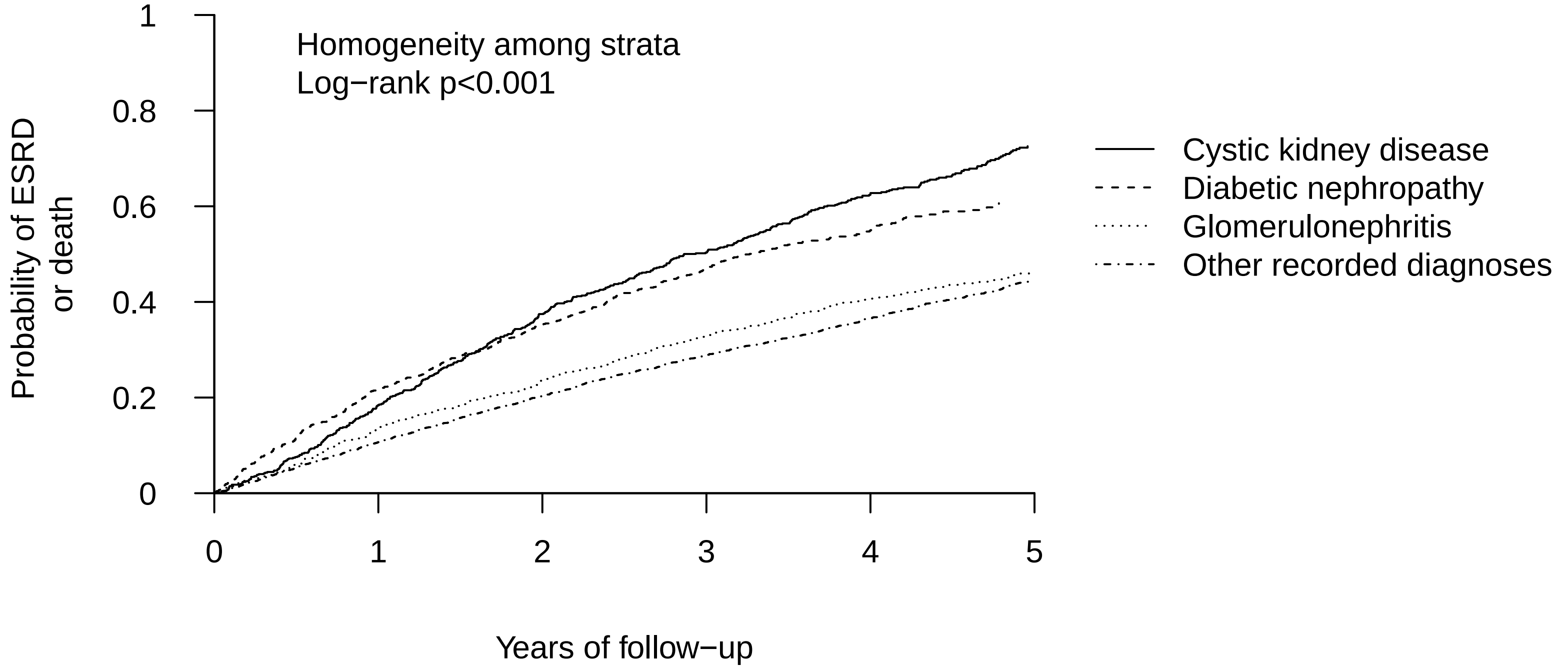

Number at risk by cause of kidney disease

Cystic kidney disease	675	549	420	328	200	81
Diabetic nephropathy	886	693	570	457	300	102
Glomerulonephritis	1049	903	797	679	502	248
Other recorded diagnoses	3380	3004	2663	2323	1648	712
