# Supplementary material for: Evaluating the Contribution of the Cause of Kidney Disease to Prognosis in CKD: Results From the Study of Heart and Renal Protection (SHARP)
Source: Am J Kidney Dis. 2014 Jul;64(1):40–8. doi: 10.1053/j.ajkd.2013.12.013 (PMC4068325; doi:10.1053/j.ajkd.2013.12.013)
Supplement: Supplementary Figure S2 (PDF) — Effect of adjustment for known risk factors on association between cause of kidney disease and ESRD. [file mmc5.pdf]

Haynes et al, AJKD, "Evaluating the Contribution of the Cause of Kidney Disease to Prognosis in CKD: Results From the Study of Heart and Renal Protection (SHARP)"

Figure S2: Effect of adjustment for known risk factors on the association between cause of kidney disease and end stage renal disease using Fine and Gray regression

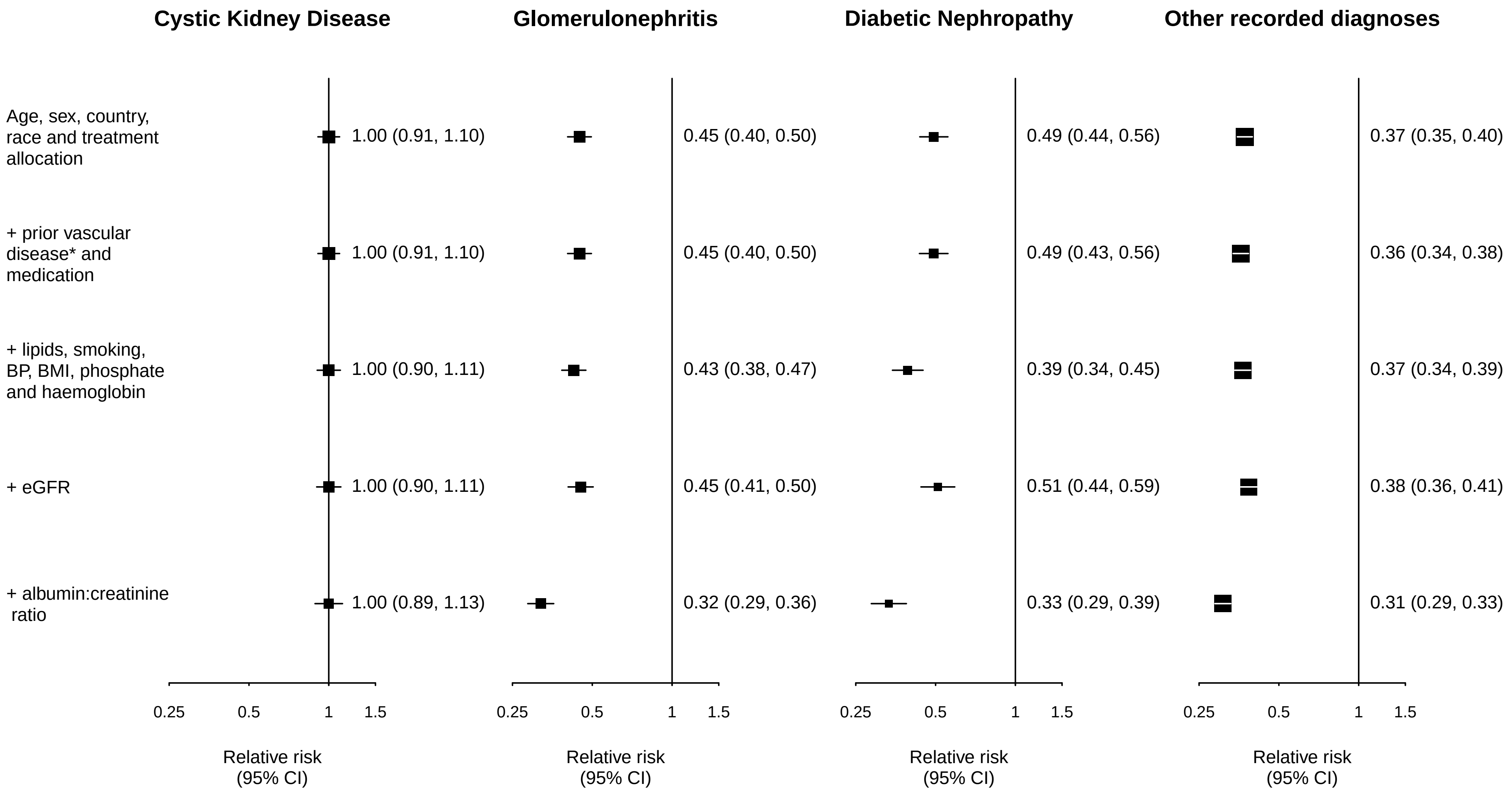

*Additional adjustment for prior diabetes has very little effect on the relative risks observed, but as the interpretation of the relative risk for the diabetic nephropathy group after adjustment for diabetes is unclear, it is not adjusted for in these analyses.
